# Supplementary material for: Local molecular and global connectomic contributions to cross-disorder cortical abnormalities
Source: Nat Commun. 2022 Aug 10;13:4682. doi: 10.1038/s41467-022-32420-y (PMC9365855; doi:10.1038/s41467-022-32420-y)
Supplement: Supplementary file 1 — Supplementary Information [file 41467_2022_32420_MOESM1_ESM.pdf]

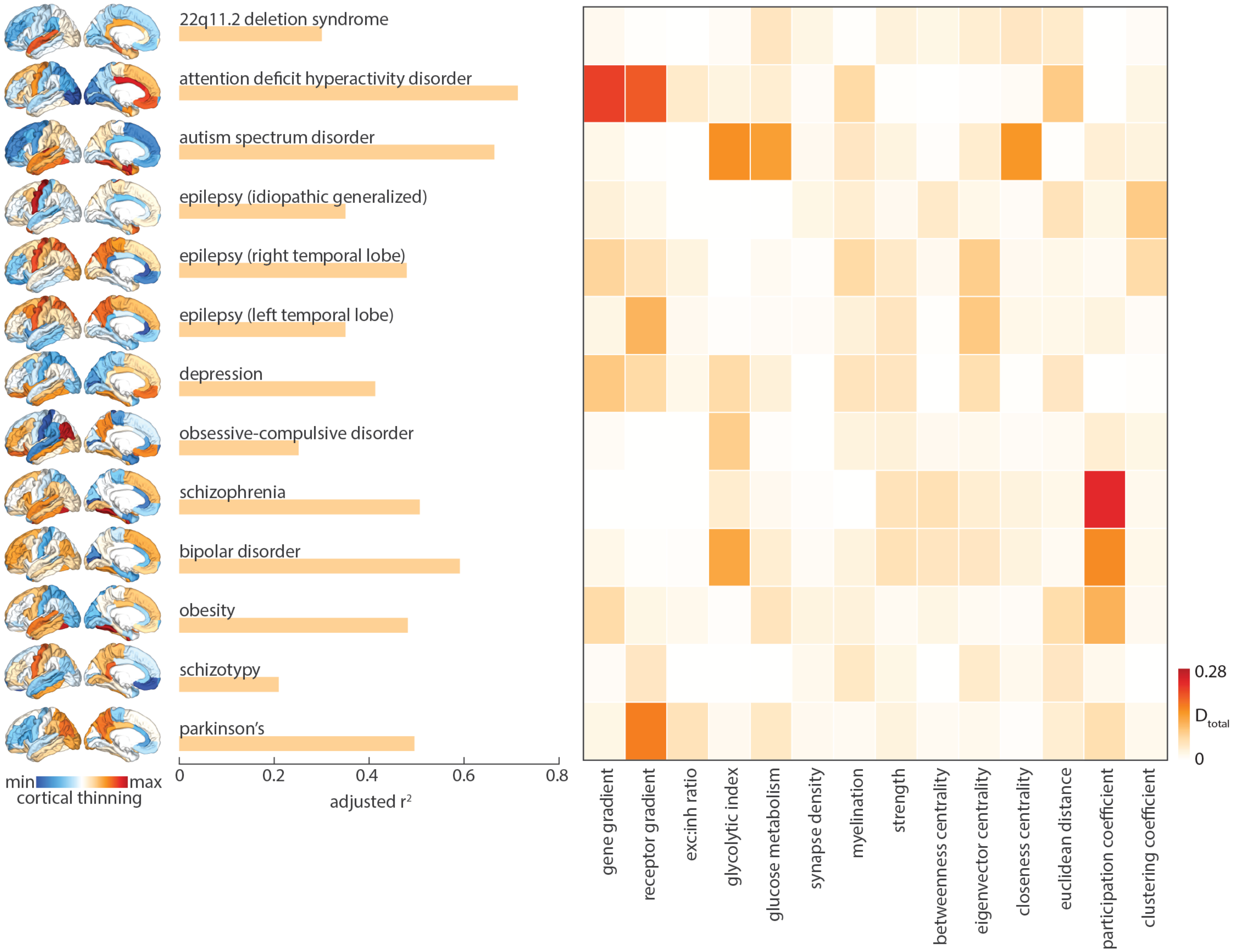

Figure S1. **Combining molecular and connectomic predictors |** Thirteen multivariate regression models were fit between the combined (molecular and connectomic) predictor set, to predict disorder-specific cortical abnormality (adjusted R^2). Dominance analysis was applied to assess the predictors that contribute most to each mode.

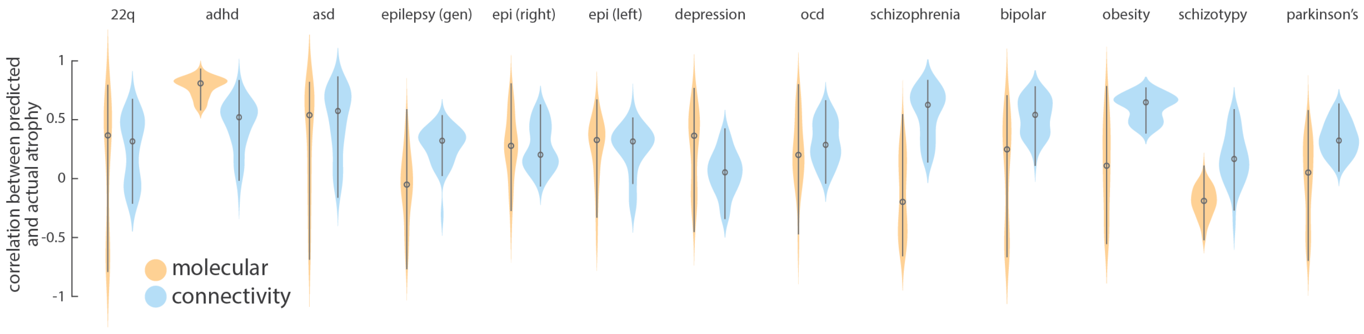

Figure S2. **Distance-dependent cross-validation |** Each model was cross-validated using a distance-dependent method. For 1 000 randomly chosen source regions, the model was fit on a training set, composed of the 75% of regions closes to the source region. Next, cortical abnormality values were predicted on the remaining 25% of regions and correlated to the empirical abnormality values. Circles represent the median and lines span the first to third quartiles.

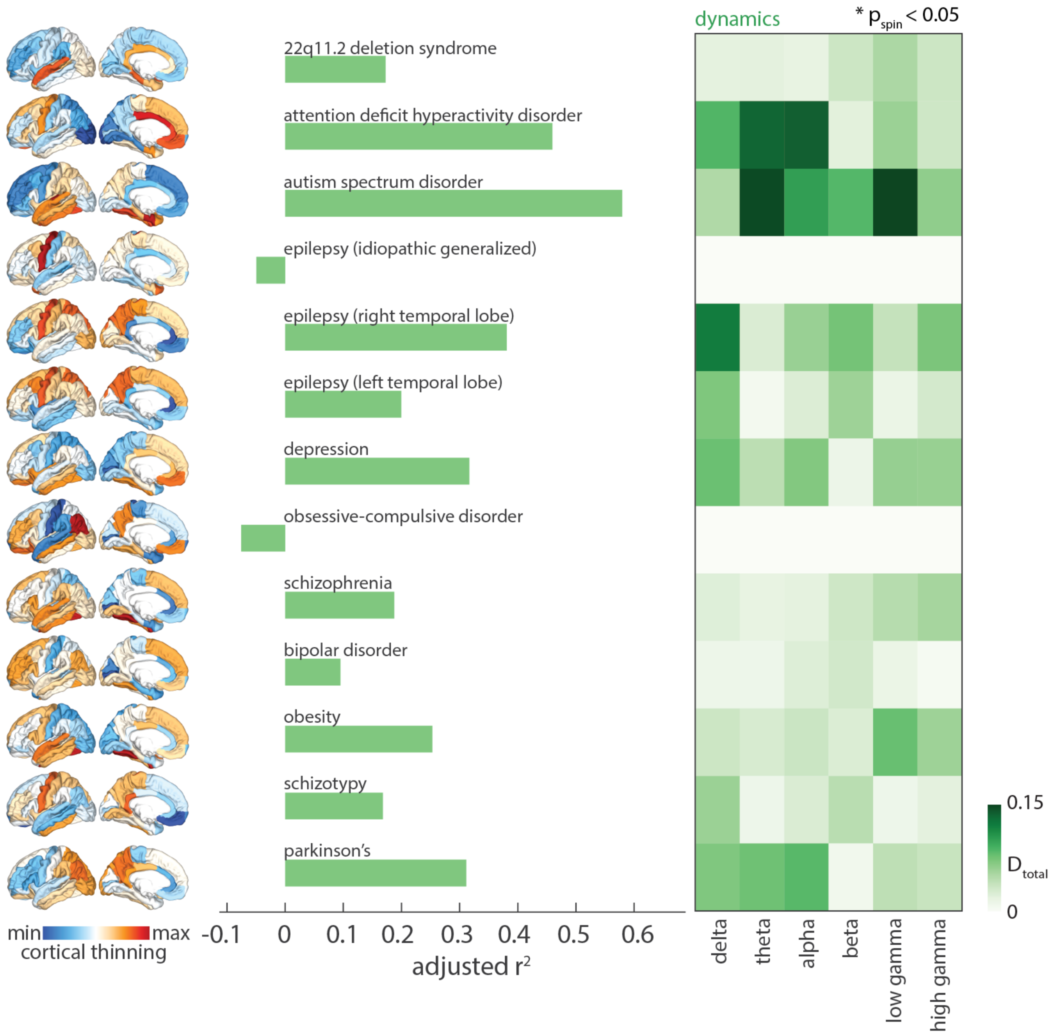

Figure S3. **Mapping MEG-derived temporal predictors to disorder-specific cortical morphology |** For each disorder, a multilinear model was fit between six MEG-derived power distributions and the abnormality pattern (adjusted R^2).

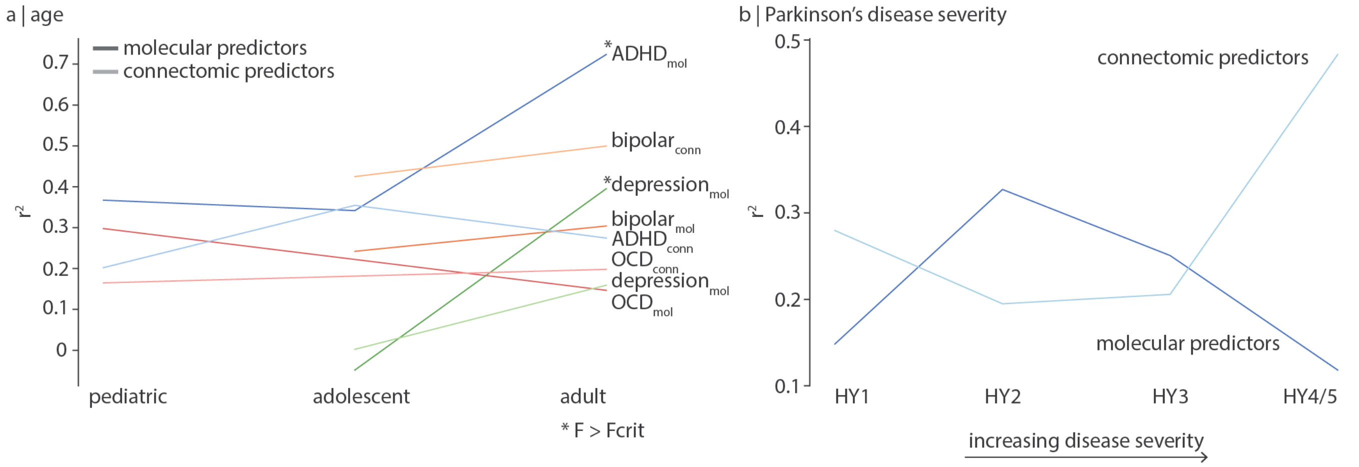

Figure S4. **Molecular and connectomic contributions to cortical abnormality change with age and disease severity |** (a) Age stratified cortical abnormality patterns are available for ADHD, bipolar disorder, depression, and OCD. We fit molecular (darker line) and connectomic (fainter line) predictors to disorder profiles across different life stages. Note that the exact age range for each category differs slightly across the four disorders. Asterisks indicate that model fit at the adult stage is significantly greater than model fit at adolescent age (or pediatric, if adolescent isn't available), against the F-test (one-sided). (b) Cortical abnormality patterns were available for four stages of disease severity in Parkinson's disease, according to the Hoehn and Yahr (HY) stages. We fit molecular (darker line) and connectomic (fainter line) predictors to cortical Parkinson's profiles at different stages of disease severity. Note that one-sided F-tests were conducted between HY4/5 and all other stages, for molecular and connectomic predictors separately, but regression models were not found to be significantly different.

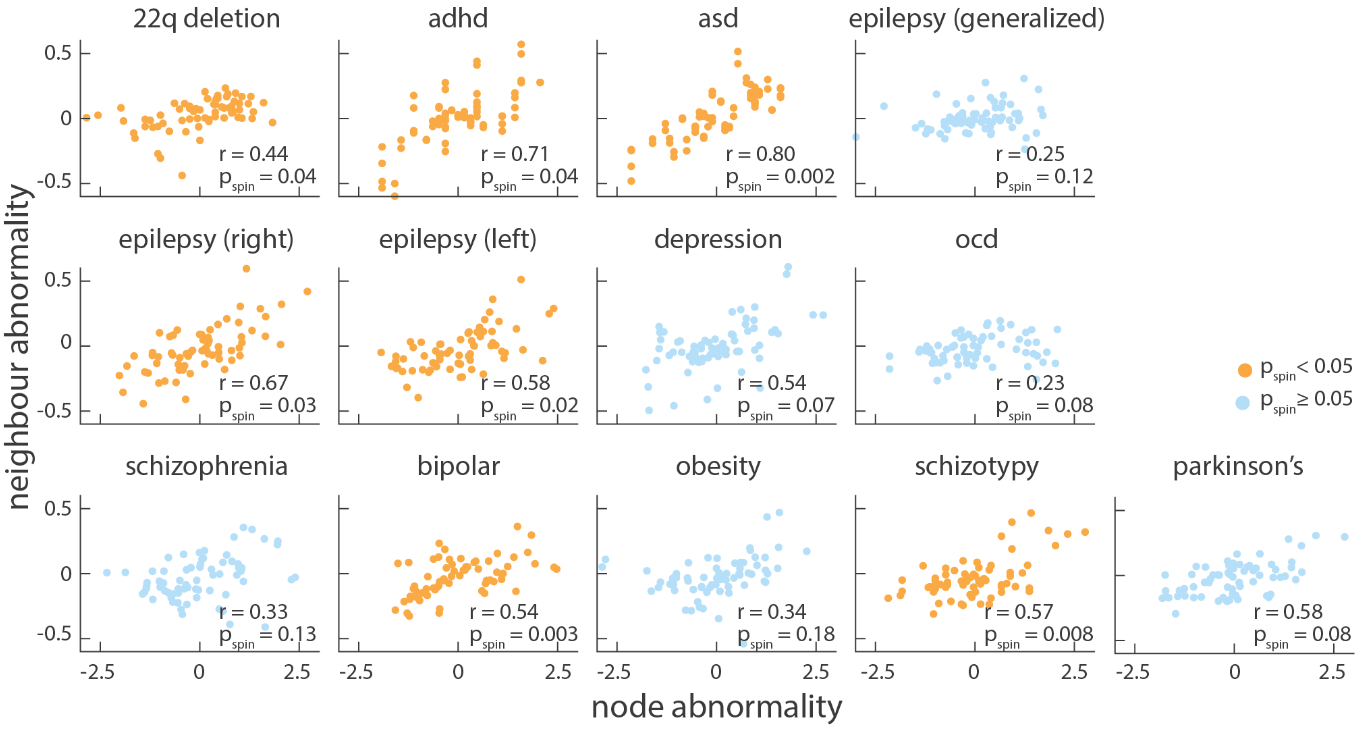

Figure S5. **Assessing network spreading disorder-specific cortical morphology using structural and functional connectivity |** A disorder whose cortical morphology demonstrates network spreading was defined as one whose regional abnormality pattern is correlated (Pearson's r) to mean neighbour abnormality, weighted by structural connectivity and functional connectivity. Yellow scatter plots indicate significant ($p_{\text{spin}} < 0.05$, two-tailed) node-neighbour correlations.

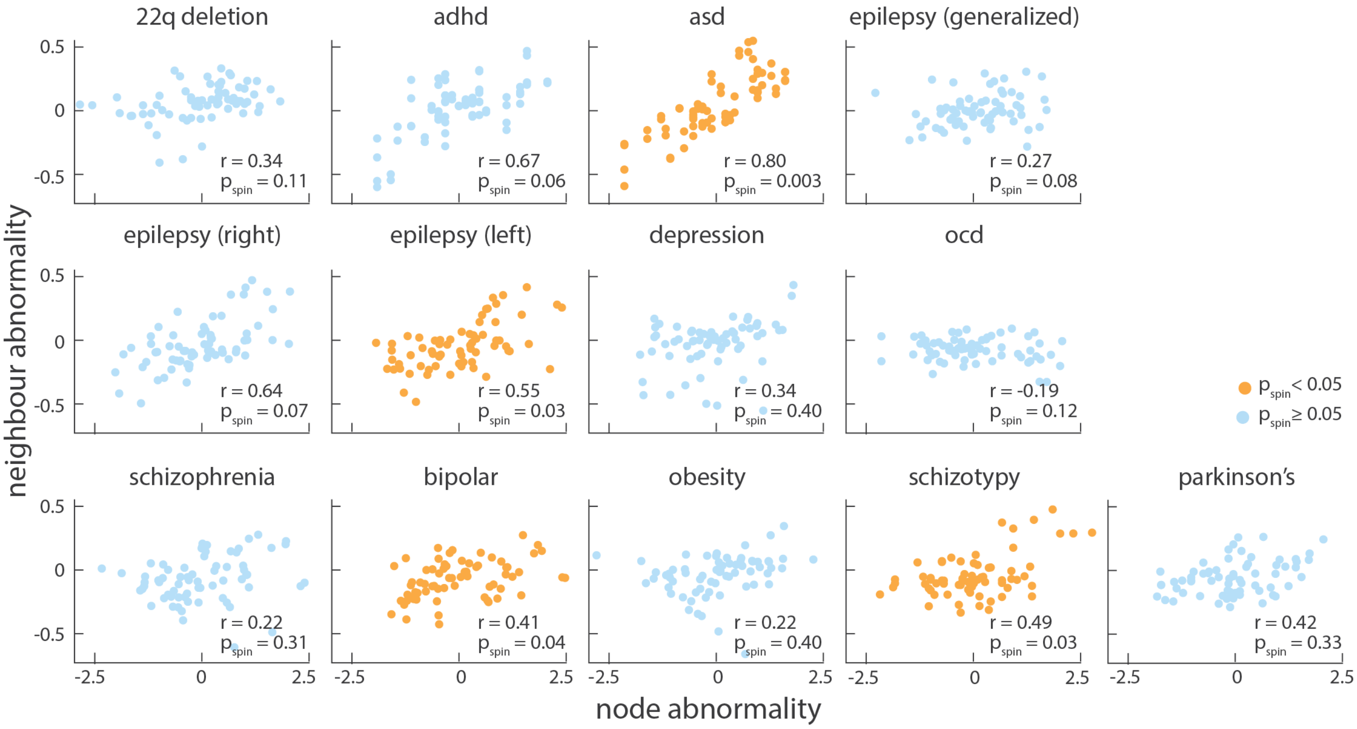

Figure S6. **Assessing network spreading disorder-specific cortical morphology using structural connectivity only** | A disorder whose cortical morphology demonstrates network spreading was defined as one whose regional abnormality pattern is correlated (Pearson's r) to mean neighbour abnormality, weighted by structural connectivity only. Yellow scatter plots indicate significant ($p_{\text{spin}} < 0.05$, two-tailed) node-neighbour correlations.

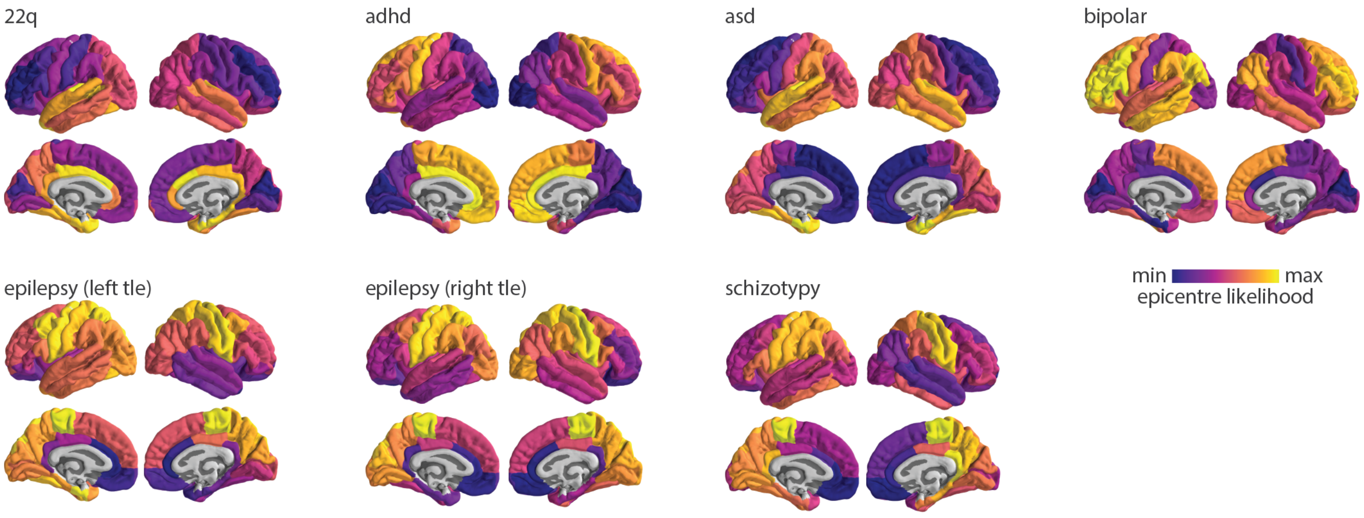

Figure S7. **Epicentre likelihood** | Epicentre likelihoods of the seven disorders that demonstrate significant correlations between node abnormality and mean sc- and fc-weighted neighbour abnormality.

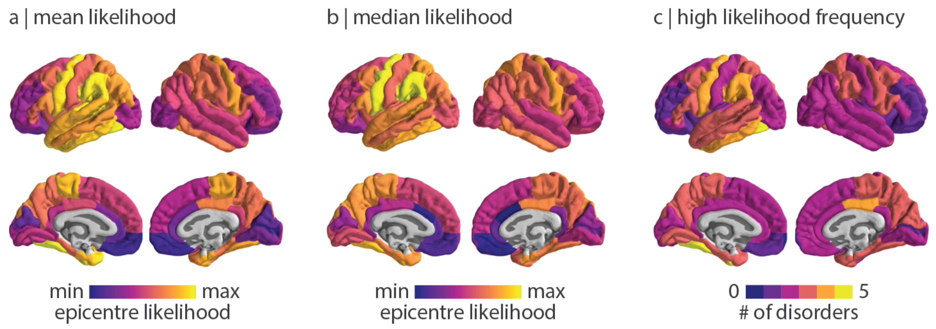

Figure S8. **Cross-disorder epicentre likelihood maps |** Epicentre likelihood was calculated for the seven disorders that show significant correlation between node and mean neighbour abnormality. To avoid biasing results, right and left temporal lobe epilepsy epicentre likelihood is represented by a single mean likelihood map (see disorder-specific epicentre likelihood maps in Fig. S7). Epicentre likelihood can be calculated as the (a) mean likelihood, (b) median likelihood (shown in Fig. 4c), and (c) frequency a brain region is in the top 50% of most likely epicentres across the included disorders.

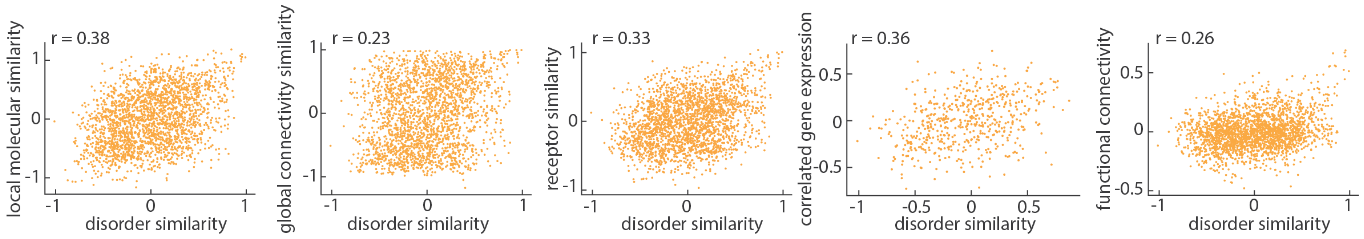

Figure S9. **Brain regions with similar molecular annotations are similarly affected across disorders |** Correlations in Fig. 5 were repeated after regressing the exponential relationship with Euclidean distance out of each similarity network (instead of applying spin-tests).

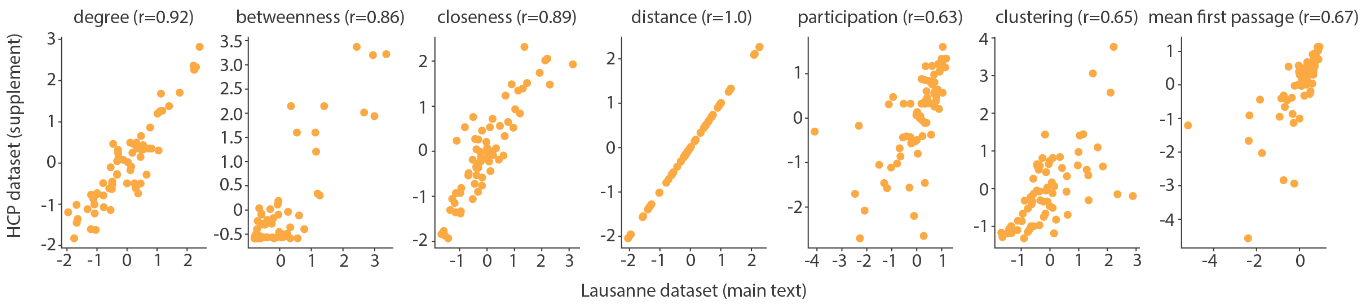

Figure S10. **Comparing connectome predictors from two different datasets |** Connectome predictors were calculated using a structural network from diffusion-spectrum MRI data from the Lausanne dataset (used in the main text) and diffusion-weighted MRI data from the Human Connectome Project (used in the supplement). Connectome predictors are consistent across datasets, acquisition parameters, and processing methodologies.

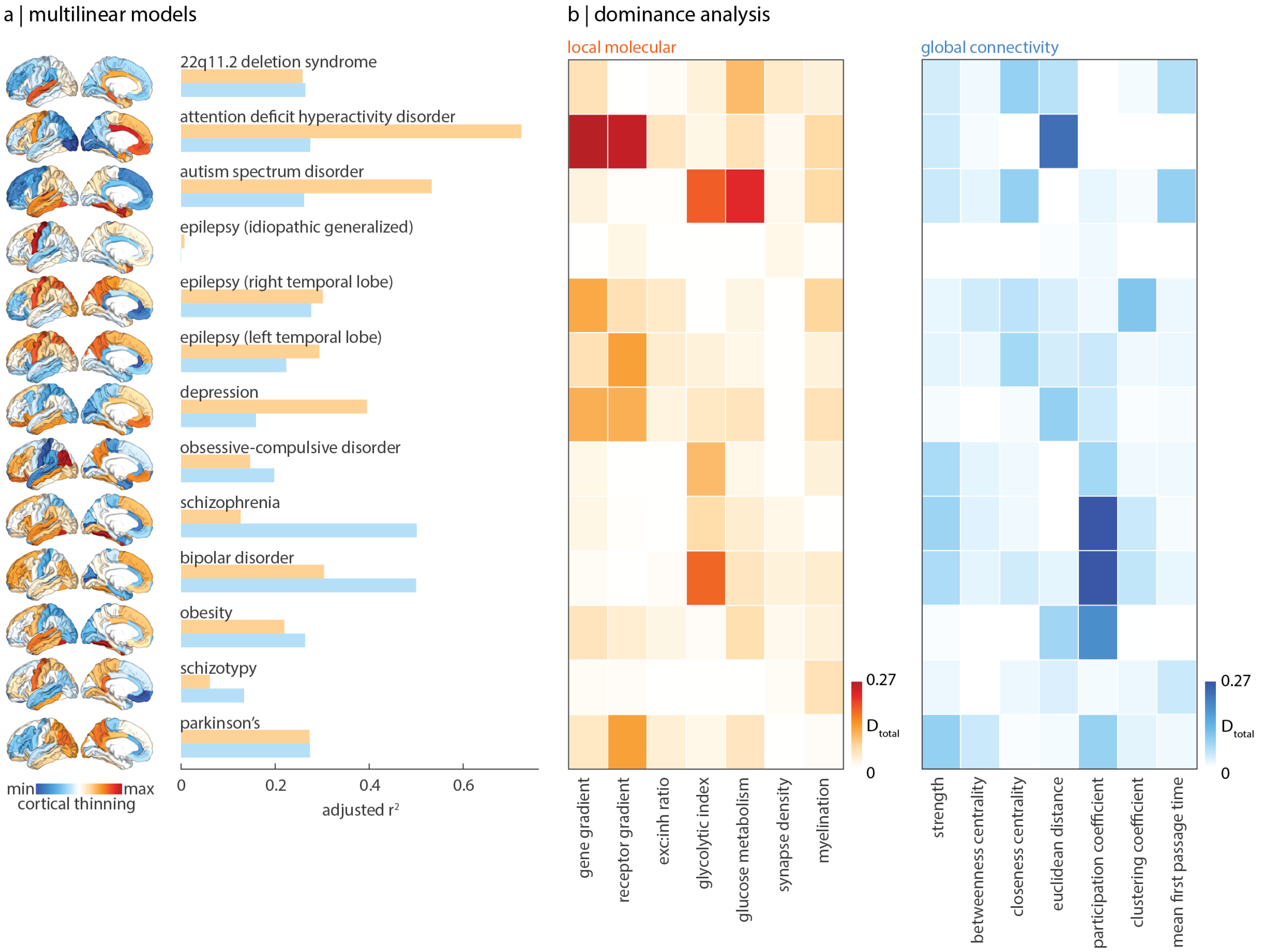

Figure S11. **Replication using HCP structural networks |** The main analysis (corresponding to Fig. 2 in the main text) was repeated using diffusion weighted MRI data from the Human Connectome Project ($N = 326$).

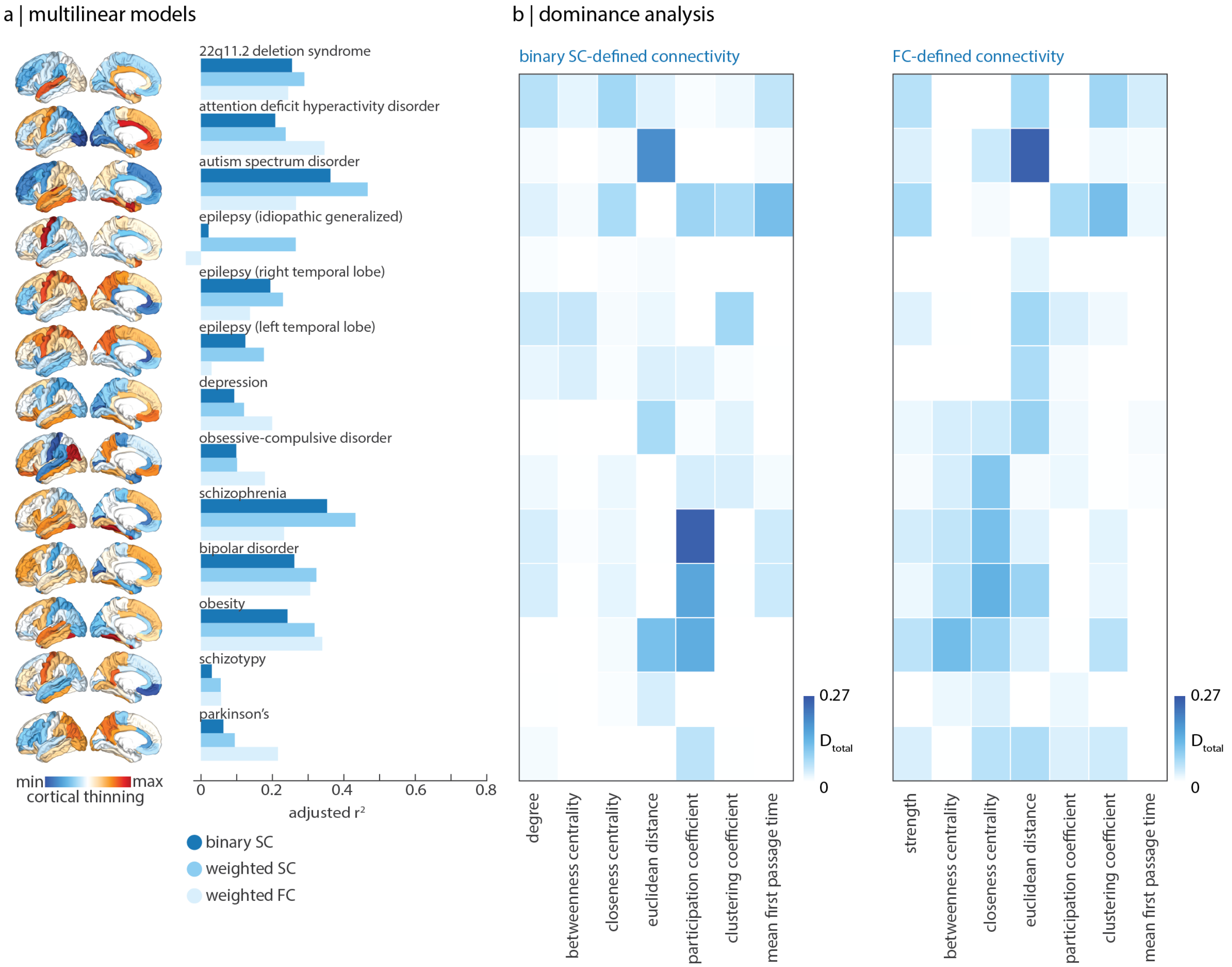

Figure S12. **Computing global network measures on different connectivity matrices |** Global network measures were calculated on the binary structural connectivity matrix as well as the weighted functional connectivity matrix. (a) Adjusted R^2 between connectivity predictors and disorder maps (left-most surfaces) when connectivity measures were calculated on the binary structural connectome (dark blue), weighted structural connectome (medium blue; used in main text analyses), and weighted functional connectome (light blue). (b) Dominance analysis for the binary structural connectome (left) and weighted functional connectome (right). Asterisks represent $p_{\text{spin}} < 0.05$.

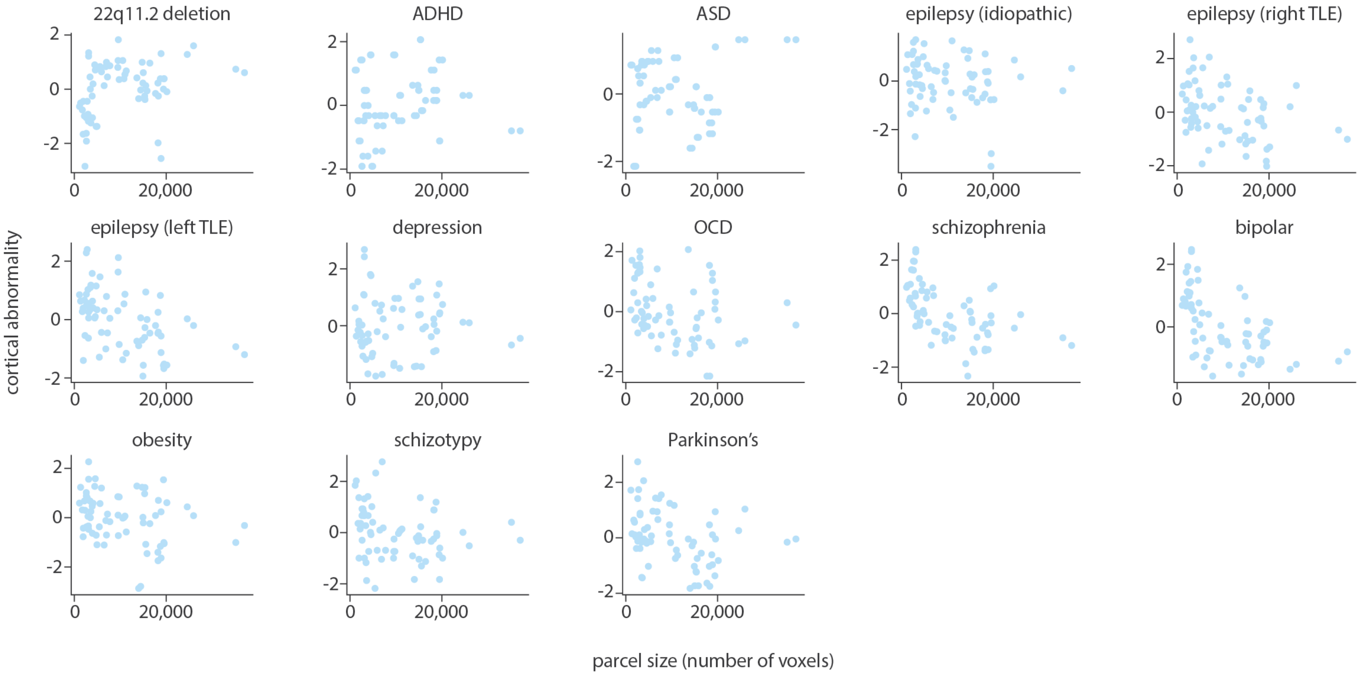

Figure S13. **Effects of parcel size on disorder profiles |** Each cortical abnormality disorder profile (y-axis) was compared to the region size of each parcel in the Desikan Killiany 68-region atlas (x-axis). Parcel size was defined as the number of MNI-152 voxels in each parcel.

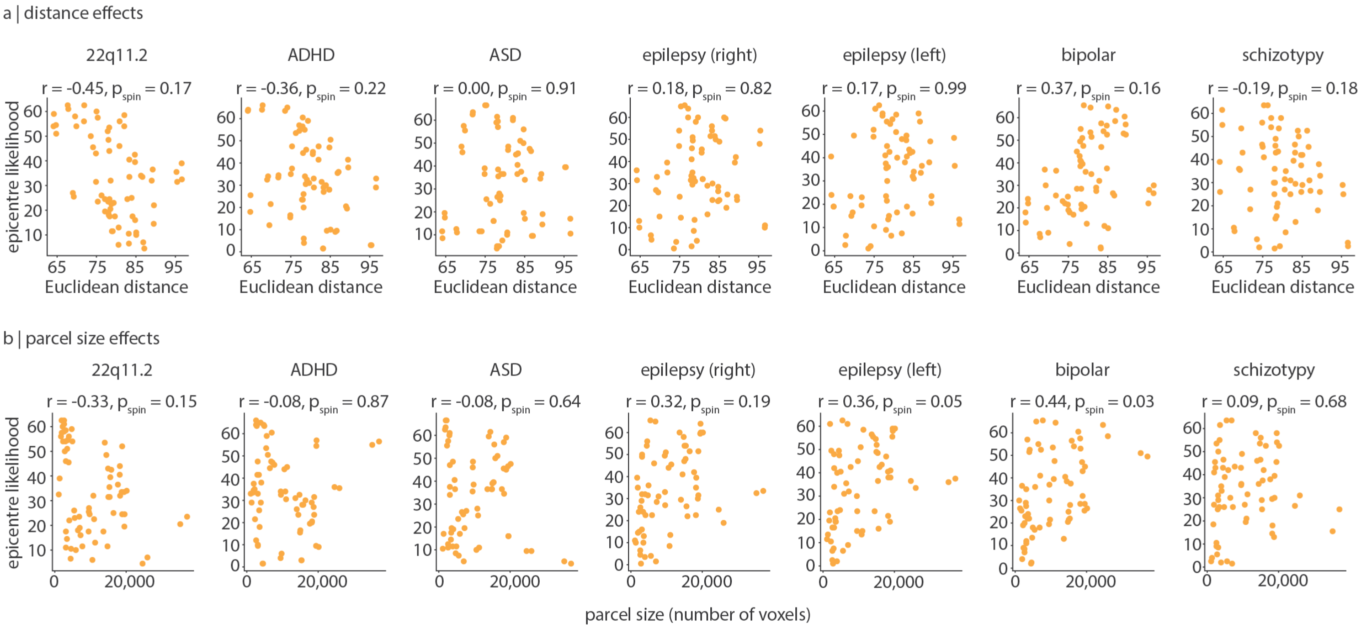

Figure S14. **Distance and parcel size effects on epicentre likelihood |** For the seven disorders that show significant correlation between node and sc-/fc-weighted neighbour abnormality, we correlated (Pearson's r, two-tailed spin-test) epicentre likelihood with (a) the average Euclidean distance between one brain region and all other brain regions, and (b) the parcel size (defined as number of voxels defined in the MNI-152 atlas) of each brain region.

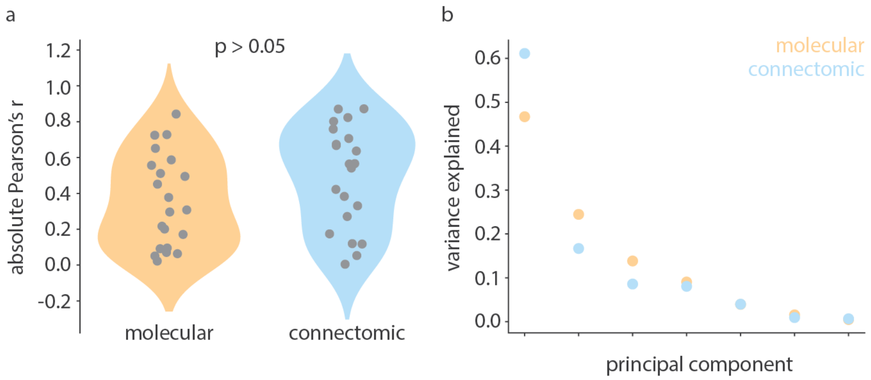

Figure S15. **Comparing molecular and connectomic predictor sets |** (a) Distributions of absolute correlations (Pearson's r) between pairs of predictors are not significantly different from one another (Welch's t-test, two-sided, $p = 0.11$). (b) PCA was applied to the region $\times$ predictor matrices for molecular and connectomic predictors separately. The first principal component of connectomic predictors explains more variance (61%) than that of molecular predictors (47%).

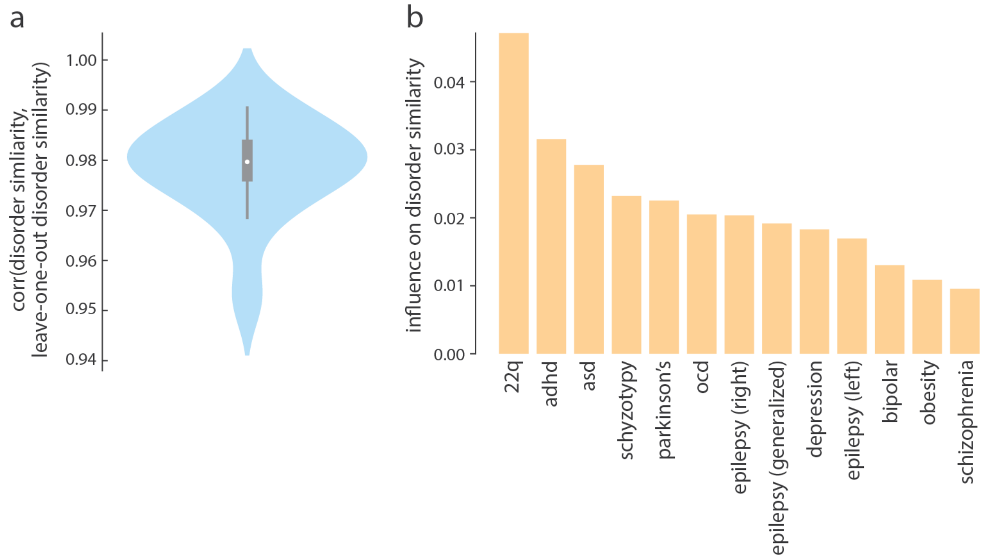

Figure S16. **Robustness of disorder similarity matrix |** (a) Disorder similarity was correlated to a version of the disorder similarity matrix, constructed by excluding a single disorder, across all thirteen disorders. The minimum correlation, computed when 22q11.2 deletion syndrome is excluded, is $r = 0.95$. Bounds of the box ($N = 13$ Pearson's correlations) represent the 1st (25%) and 3rd (75%) quartiles, the centre circle represents the median, and whiskers represent the minima and maxima of the distribution. (b) The influence that each disorder has on the disorder similarity matrix is calculated as the difference between 1 and the correlation coefficient calculated in (a).
