# Supplementary material for: Local molecular and global connectomic contributions to cross-disorder cortical abnormalities
Source: Nat Commun. 2022 Aug 10;13:4682. doi: 10.1038/s41467-022-32420-y (PMC9365855; doi:10.1038/s41467-022-32420-y)
Supplement: Supplementary file 3 — Reporting Summary [file 41467_2022_32420_MOESM3_ESM.pdf]

nature portfolio

Corresponding author(s): Bratislav Misic

Last updated by author(s): Jul 4, 2022

Reporting Summary

Nature Portfolio wishes to improve the reproducibility of the work that we publish. This form provides structure for consistency and transparency in reporting. For further information on Nature Portfolio policies, see our Editorial Policies and the Editorial Policy Checklist.

Statistics

For all statistical analyses, confirm that the following items are present in the figure legend, table legend, main text, or Methods section.

n/a	Confirmed	
☐	☒	The exact sample size (n) for each experimental group/condition, given as a discrete number and unit of measurement
☒	☐	A statement on whether measurements were taken from distinct samples or whether the same sample was measured repeatedly
☐	☒	The statistical test(s) used AND whether they are one- or two-sided<br>*Only common tests should be described solely by name; describe more complex techniques in the Methods section.*
☒	☐	A description of all covariates tested
☐	☒	A description of any assumptions or corrections, such as tests of normality and adjustment for multiple comparisons
☐	☒	A full description of the statistical parameters including central tendency (e.g. means) or other basic estimates (e.g. regression coefficient) AND variation (e.g. standard deviation) or associated estimates of uncertainty (e.g. confidence intervals)
☐	☒	For null hypothesis testing, the test statistic (e.g. F, t, r) with confidence intervals, effect sizes, degrees of freedom and P value noted<br>*Give P values as exact values whenever suitable.*
☒	☐	For Bayesian analysis, information on the choice of priors and Markov chain Monte Carlo settings
☒	☐	For hierarchical and complex designs, identification of the appropriate level for tests and full reporting of outcomes
☐	☒	Estimates of effect sizes (e.g. Cohen's d, Pearson's r), indicating how they were calculated

Our web collection on statistics for biologists contains articles on many of the points above.

Software and code

Policy information about availability of computer code

Data collection	enigmatoolbox v1.1.1 (https://github.com/MICA-MNI/ENIGMA) was used for fetching ENIGMA data.<br>MEG data was processed using the open software toolbox Brainstorm v220420.<br>HCP structural data was processed using MRtrix3 v3.0.0
Data analysis	Data analysis was conducted using Python v3.8.10, standard Python packages and neuromaps v0.0.1 (https://netneurolab.github.io/neuromaps/).

For manuscripts utilizing custom algorithms or software that are central to the research but not yet described in published literature, software must be made available to editors and reviewers. We strongly encourage code deposition in a community repository (e.g. GitHub). See the Nature Portfolio guidelines for submitting code & software for further information.

Data

Policy information about availability of data

All manuscripts must include a data availability statement. This statement should provide the following information, where applicable:

- Accession codes, unique identifiers, or web links for publicly available datasets
- A description of any restrictions on data availability
- For clinical datasets or third party data, please ensure that the statement adheres to our policy

All data used in the present report is openly available at https://github.com/netneurolab/hansen_crossdisorder_vulnerability. More specifically, ENIGMA datasets are available through the ENIGMA consortium and the ENIGMA toolbox (https://github.com/MICA-MNI/ENIGMA). The Lausanne dataset is available at https://zenodo.org/record/2872624#.XOJqE99fhmM. The HCP dataset is available at https://db.humanconnectome.org/. Molecular predictors are available as volumetric images in the neuromaps toolbox (https://netneurolab.github.io/neuromaps/). The Allen Human Brain Atlas is available at https://human.brain-map.org/.

Field-specific reporting

Please select the one below that is the best fit for your research. If you are not sure, read the appropriate sections before making your selection.

- [x] Life sciences
- [ ] Behavioural & social sciences
- [ ] Ecological, evolutionary & environmental sciences

For a reference copy of the document with all sections, see nature.com/documents/nr-reporting-summary-flat.pdf

Life sciences study design

All studies must disclose on these points even when the disclosure is negative.

Sample size	Sample sizes of individual datasets were not chosen as only open-source data was used. ENIGMA datasets were chosen as the maximum number of disorders with open data (as far as the authors were aware at the time of the analyses). The number of molecular predictors was selected as the maximum number of non-redundant open molecularly-relevant annotations in the brain. Connecomic predictors were likely chosen as the maximum number of non-redundant, easily interpretable, graph theoretic measures of the connectome.
Data exclusions	Non adult participants were excluded.
Replication	Analyses were repeated using a different structural and functional connectivity dataset (HCP), for which the replication was successful.
Randomization	Randomization was not performed because participants were not placed into experimental groups (except for the case and control groups, which are defined based on diagnosis).
Blinding	Blinding is not relevant to this study because participants were not placed into experimental groups.

Reporting for specific materials, systems and methods

We require information from authors about some types of materials, experimental systems and methods used in many studies. Here, indicate whether each material, system or method listed is relevant to your study. If you are not sure if a list item applies to your research, read the appropriate section before selecting a response.

Materials & experimental systems

n/a	Involved in the study	
x		Antibodies
x		Eukaryotic cell lines
x		Palaeontology and archaeology
x		Animals and other organisms
	x	Human research participants
x		Clinical data
x		Dual use research of concern

Methods

n/a	Involved in the study	
x		ChIP-seq
x		Flow cytometry
	x	MRI-based neuroimaging

Human research participants

Policy information about studies involving human research participants

Population characteristics	Structural and functional data were collected at the Department of Radiology, University Hospital Center and University of Lausanne, on n=70 healthy young adults (16 females, 25.3+/- 4.9 years).
Recruitment	NA
Ethics oversight	Informed consent was obtained from all participants and the protocol was approved by the Ethics Committee of Clinical Research of the Faculty of Biology and Medicine, University of Lausanne.

Note that full information on the approval of the study protocol must also be provided in the manuscript.

Magnetic resonance imaging

Experimental design

Design type	Resting-state fMRI and diffusion-weighted MRI
Design specifications	The session protocol was comprised of (1) a magnetization-prepared rapid acquisition gradient echo (MPRAGE) sequence sensitive to white/gray matter contrast (1-mm in-plane resolution, 1.2-mm slice thickness), (2) a DSI sequence (128 diffusion-weighted volumes and a single b0 volume, maximum b-value 8,000 s/mm2, 2.2x2.2x3.0 mm voxel size),

and (3) a gradient echo EPI sequence sensitive to BOLD contrast (3.3-mm in-plane resolution and slice thickness with a 0.3-mm gap, TR 1,920 ms, resulting in 280 images per participant). During the fMRI scan, participants were not engaged in any overt task, and the scan was treated as eyes-open resting-state fMRI (rs-fMRI).

Behavioral performance measures: No behavioural measures were recorded during the fMRI runs.

Acquisition

Imaging type(s): functional and diffusion

Field strength: 3T

Sequence & imaging parameters: The session protocol was comprised of (1) a magnetization-prepared rapid acquisition gradient echo (MPRAGE) sequence sensitive to white/gray matter contrast (1-mm in-plane resolution, 1.2-mm slice thickness), (2) a DSI sequence (128 diffusion-weighted volumes and a single b0 volume, maximum b-value 8,000 s/mm2, 2.2x2.2x3.0 mm voxel size), and (3) a gradient echo EPI sequence sensitive to BOLD contrast (3.3-mm in-plane resolution and slice thickness with a 0.3-mm gap, TR 1,920 ms, resulting in 280 images per participant). During the fMRI scan, participants were not engaged in any overt task, and the scan was treated as eyes-open resting-state fMRI (rs-fMRI).

Area of acquisition: Whole-brain.

Diffusion MRI: [x] Used [ ] Not used

Parameters: 128 diffusion-weighted volumes and a single b0 volume, maximum b-value 8,000 s/mm2, 2.2x2.2x3.0 mm voxel size

Preprocessing

Preprocessing software: Connectome Mapper pipeline, Freesurfer

Normalization: For further details about data processing, please refer to https://doi.org/10.5281/zenodo.2872624

Normalization template: Lausanne anatomical atlas (subdivision of the Desikan-Killiany atlas)

Noise and artifact removal: For further details about data processing, please refer to https://doi.org/10.5281/zenodo.2872624

Volume censoring: For further details about data processing, please refer to https://doi.org/10.5281/zenodo.2872624

Statistical modeling & inference

Model type and settings: Functional and structural connectomes were used for comparison with ENIGMA derived disease profiles.

Effect(s) tested: Correlation between FC and disorder similarity (Pearson's r), and graph measurements were taken on the SC and FC matrices.

Specify type of analysis: [x] Whole brain [ ] ROI-based [ ] Both

Statistic type for inference (See Eklund et al. 2016): NA

Correction: NA

Models & analysis

n/a	Involved in the study
[ ]	[x] Functional and/or effective connectivity
[ ]	[x] Graph analysis
[ ]	[x] Multivariate modeling or predictive analysis

Functional and/or effective connectivity: Pearson's correlation

Graph analysis: Main graph analyses were conducted on the weighted structural connectome but was then repeated on the binary structural connectome and the weighted functional connectome.

Multivariate modeling and predictive analysis: Multilinear regression models were fit between biological/connectivity predictors (independent variable) and 13 disorder profiles (dependent variable), where brain regions are the observations. Adjusted R^2 was used to estimate goodness of fit and models were cross-validated using a distance-dependent method first introduced in Hansen 2021 Nat Hum Behav.
